# Impairment of toll-like receptors 2 and 4 leads to compensatory mechanisms after sciatic nerve axotomy

**DOI:** 10.1186/s12974-016-0579-6

**Published:** 2016-05-24

**Authors:** C. M. Freria, D. Bernardes, G. L. Almeida, G. F. Simões, G. O. Barbosa, A. L. R. Oliveira

**Affiliations:** Department of Structural and Functional Biology, Institute of Biology, University of Campinas, Campinas, Sao Paulo Brazil

**Keywords:** Pheripheral nerve injury, Axotomy, Toll-like receptors and functional recovery

## Abstract

**Background:**

Peripheral nerve injury results in retrograde cell body-related changes in the spinal motoneurons that will contribute to the regenerative response of their axons. Successful functional recovery also depends on molecular events mediated by innate immune response during Wallerian degeneration in the nerve microenvironment. A previous study in our lab demonstrated that TLR 2 and 4 develop opposite effects on synaptic stability in the spinal cord after peripheral nerve injury. Therefore, we suggested that the better preservation of spinal cord microenvironment would positively influence distal axonal regrowth. In this context, the present work aimed to investigate the influence of TLR2 and TLR4 on regeneration and functional recovery after peripheral nerve injury.

**Methods:**

Eighty-eight mice were anesthetized and subjected to unilateral sciatic nerve crush (C3H/HeJ, n = 22, C3H/HePas, *n* = 22; C57Bl6/J, *n* = 22 and TLR2^−/−^, *n* = 22). After the appropriate survival times (3, 7, 14 days, and 5 weeks), all mice were killed and the sciatic nerves and tibialis cranialis muscles were processed for immunohistochemistry and transmission electron microscopy (TEM). Gait analysis, after sciatic nerve crushing, was performed in another set of mice (minimum of *n* = 8 per group), by using the walking track test (CatWalk system).

**Results:**

TLR4 mutant mice presented greater functional recovery as well as an enhanced p75^NTR^ and neurofilament protein expression as compared to the wild-type strain. Moreover, the better functional recovery in mutant mice was correlated to a greater number of nerve terminal sprouts. Knockout mice for TLR2 exhibited 30 % greater number of degenerated axons in the distal stump of the sciatic nerve and a decreased p75^NTR^ and neurofilament protein expression compared to the wild type. However, the absence of TLR2 receptor did not influence the overall functional recovery. End-point equivalent functional recovery in transgenic mice may be a result of enhanced axonal diameter found at 2 weeks after lesion.

**Conclusions:**

Altogether, the present results indicate that the lack of TLR2 or the absence of functional TLR4 does affect the nerve regeneration process; however, such changes are minimized through different compensatory mechanisms, resulting in similar motor function recovery, as compared to wild-type mice. These findings contribute to the concept that innate immune-related molecules influence peripheral nerve regeneration by concurrently participating in processes taking place both at the CNS and PNS.

**Electronic supplementary material:**

The online version of this article (doi:10.1186/s12974-016-0579-6) contains supplementary material, which is available to authorized users.

## Background

Peripheral nerve injury (PNI) triggers a series of local responses in the damaged microenvironment, which in turn influence the regenerative potential of axotomized neurons. Rapid and efficient clearance of myelin debris is essential and critical for successful nerve regeneration. In addition, functional recovery depends on a number of factors, but is directly affected by the course of cellular and molecular events of Wallerian degeneration (WD) [1], mostly at the nerve distal stump. WD is characterized by inflammation and extensive fragmentation of axon debris that must be cleared by phagocytosis. Schwann cells and macrophages are involved in myelin phagocytosis [2], and the efficiency of the phagocytic process is linked to the expression of immune-related receptors [3, 4].

The inflammatory reaction at the site of injury is firstly elicited by Schwann cells and further by recruited macrophages [5]. Activated Schwann cells, in the distal nerve stump, express many inflammatory mediators such as cytokines, chemokines, interleukins, tumor necrosis factor alpha (TNF-α), and inducible nitric oxide synthase (iNOS). Within hours to days, this network is further amplified by recruited macrophages [6]. However, it is not completely understood how Schwann cells orchestrate the initial response to axotomy, and relatively recent studies have shown that expression of toll-like receptors (TLRs) may be involved in the process [7].

TLRs are found in the brain, being expressed by microglia, astrocytes, oligodendrocytes, and neurons [8]. They are pattern recognition receptors activated by stereotyped ligands, usually secreted by microorganisms or lesioned cells. It is possible that such ligands are also present in the damaged nerve, thereby recruiting macrophages into such microenvironment, contributing to the clearance of axon and myelin [3, 4, 9]. Such innate immunological response appears to be helpful in preparing the lesioned peripheral nerve tissue to support axonal regeneration, by removing inhibitory myelin debris, and by upregulating local neurotrophic factors production. Therefore, the axonal regeneration is dependent on microenvironment that favors regrowth [10]. Findings have shown that deficiency in TLR signaling delays macrophage recruitment/activation, myelin debris clearance, axonal regeneration, and locomotor recovery after sciatic nerve lesion [9, 11]. On the other hand, activation of TLR2 or TLR4 accelerated phagocytosis of myelin debris and recovery of peripheral nerve function [11]. Wallerian degeneration progression, as well as macrophage influx to the lesioned peripheral nerve, however, seems to be dependent on different factors, including the extension of the lesion. In this sense, by the use of a microcrush lesion, it has been demonstrated that TLR signaling is crucial for WD progression as well as for axonal regrowth [11].

We have recently demonstrated that TLR 2 and 4 develop opposite effects on synaptic stability in the spinal cord after peripheral nerve injury. In this sense, the deficiency of TLR4 contributes to exacerbated synaptic retraction of terminals in contact to lesioned motoneurons after peripheral injury. On the contrary, the absence of TLR2 leads to synaptic preservation after peripheral axotomy, also correlating with a decrease of astrogliosis and presence of pro-inflammatory interleukin mRNA [12]. Synaptic retraction and re-apposition of pre-synaptic terminals following injury have been correlated to the ability of lesioned neurons to produce a significant regenerative response. Selectivity towards maintenance of inhibitory inputs and retraction of excitatory synapses is in line with this concept and has been shown earlier [13].

The present study investigated the regenerative outcome of TLR 2- and 4-deficient mice following peripheral nerve lesion, based on previously reported spinal cord input changes in such stains [12]. The motor behavior was analyzed using the most sensitive walking track test apparatus available with emphasis on the paw pressure recovery relating directly to muscle reinnervation and strength. The data herein reveals that absence of functional TLR 4 gives an adaptive advantage in terms of muscle strength recovery, possibly due to a greater establishment of neuromuscular junctions, reducing the size of motor units. Lack of TLR 2, result in faster clearance of axon debris, contributing to a greater regenerated axon mean diameter following lesion. Overall, such compensatory mechanisms led to similar motor function recovery, as compared to wild-type mice. Our data indicate that TLR may be interesting future therapy targets to treat and enhance peripheral nerve lesion.

## Methods

One hundred and twenty four adult mice from different strains, namely C3H/HeJ (TLR4 mutant, *n* = 30) and their wild-type counterparts, C3H/HePas (wild type, *n* = 30), TLR2^-/-^ (knockout *n* = 32) and C57BL/6 J (wild type, *n* = 32), 6–8 weeks old, were obtained from the Multidisciplinary Centre of Biological Investigation (CEMIB/Unicamp). During the experiments, they were housed under a 12-h light/dark cycle with free access to food and water. The Institutional Committee for Ethics in Animal Use approved the study (CEUA/IB/Unicamp, no. 1656-1 and 2104-1), and the experiments were carried out in accordance with the guidelines of the National Council for the Control of Animal Experimentation (CONCEA).

### Surgical procedures and tissue preparation

Mice were anesthetized with a mixture of Kensol (xylazine, Köning, 10 mg/kg) and Vetaset (Cetamin, Fort Dodge, 50 mg/kg, 1:1, 0.12 mL/25 g, i.p.) and were subjected to left sciatic nerve crush at the level of the greater sciatic notch. The left sciatic nerve was exposed at mid-thigh level and crushed at full pressure for 60 s with a pair of jewelers forceps (no. 4), thereby allowing complete transection of neural fibers without breaking the epineurium according to [14]. Muscle and skin layers were sutured, and the animals were kept in the animal housing facility for appropriate survival times (3, 7, 14 days, and 5 weeks) as showed at Table 1.

**Table 1:** Experimental groups used in different time points and techniques

Mouse strain	*n*	Survival time post-surgery	Technique
C3H/HePas	3	3 days	Immunohistochemistry - sciatic nerve
	3	7 days	Immunohistochemistry - sciatic nerve
	13	2 weeks	Immunohistochemistry and TEM - sciatic nerve
	3	5 weeks	Immunohistochemistry - *m. tibialis cranialis*
	8	8 weeks	Walking track test analysis
C3H/HeJ	3	3 days	Immunohistochemistry - sciatic nerve
	3	7 days	Immunohistochemistry - sciatic nerve
	13	2 weeks	Immunohistochemistry and TEM - sciatic nerve
	3	5 weeks	Immunohistochemistry - *m. tibialis cranialis*
	8	8 weeks	Walking track test analysis
C57BL/6 J	3	3 days	Immunohistochemistry - sciatic nerve
	3	7 days	Immunohistochemistry - sciatic nerve
	13	2 weeks	Immunohistochemistry and TEM - sciatic nerve
	3	5 weeks	Immunohistochemistry - *m. tibialis cranialis*
	10	8 weeks	Walking track test analysis
TLR2^−/−^	3	3 days	Immunohistochemistry - sciatic nerve
	3	7 days	Immunohistochemistry - sciatic nerve
	13	2 weeks	Immunohistochemistry and TEM - sciatic nerve
	3	5 weeks	Immunohistochemistry - *m. tibialis cranialis*
	10	8 weeks	Walking track test analysis

*TEM* transmission electron microscopy

All mice were sacrificed with an overdose of anesthesia, subjected to transcardiac perfusion with 0.1 M PBS (20 ml, pH 7.4) and then fixed with 4 % formaldehyde in PBS for immunohistochemistry or with 2.5 % glutaraldehyde and 1.0 % paraformaldehyde in phosphate buffer (PB) pH 7.4 for electron microscopy. Ipsi- and contralateral sciatic nerves were dissected out, cryoprotected with 30 % sucrose and frozen in isopentane at −40 °C for immunohistochemistry or embedded in resin (Durcupan, ACS) for electron microscopy. The tibialis cranialis muscle were removed cryoprotected with 20 % sucrose and frozen in isopentane at −60 °C for immunohistochemistry.

### Immunohistochemistry

Nerves were longitudinally sectioned (12-μm thick) and the tibialis cranialis muscles were cut horizontally (18 μm). All sections were transferred to gelatin-coated slides and blocked in Tris-buffered saline with Triton X-100 (TBS-T) with 3 % BSA at room temperature for 1 h. The sciatic nerve sections, from mice submitted to nerve crushing, were incubated overnight at 4 °C in a moist chamber with goat anti-p75NTR (1:200, Santa Cruz Biotechnology, CA, USA), mouse anti-neurofilament (1:300, Chemicon, Temecula, CA, USA) or rabbit anti-Iba1 (1:700; Wako Chemicals USA, Richmond, VA, USA) diluted in TBS-T with 1 % BSA. After a further set of washes in TBS-T, the sections were incubated with Cy3-conjugated or Cy2-conjugated secondary antibodies (1:500; Jackson ImmunoResearch, Bar Harbor, ME, USA) for 1 h in a moist chamber at room temperature. The tibialis cranialis muscles were incubated for 1 h at room temperature in a moist chamber with α-bungarotoxin (1:500 tetramethylrhodamine conjugate; Life Technologies-USA) diluted in TBS-T with 1 % BSA. The slides were then rinsed in TBS-T, mounted in a mix of glycerol and PBS (3:1 ratio). The slides of nerves were viewed under a fluorescence microscope (Eclipse TS100, Nikon, Tokyo, Japan) equipped with a digital camera (DXM1200F; Nikon, Tokyo, Japan). The muscle tissue from the tibialis cranialis muscle was analyzed by using the optical system second harmonic generation (SHC) in a confocal Zeiss LSM780-NLO system (Carl Zeiss AG, Germany).

#### Measurement of reactive macrophage in the distal and proximal stump of sciatic nerve

For quantitative measurements of reactive macrophages, three representative sections from distal and proximal stumps at the ipsilateral side of the lesion (*n* = 3 for each group) were evaluated. The images, at a final magnification of ×20, were taken using identical settings. Quantification was performed with the enhance contrast and density slicing feature of ImageJ software (version 1.33, National Institutes of Health, USA). The integrated density of pixels was measured over the entire image surface. The average of the integrated density of pixels was calculated for each entire image (each slides) and then for each group and compared among them.

#### Estimation of neuromuscular junctions after sciatic nerve regeneration

Sections in the longitudinal plane from ipsi and contralateral tibialis cranialis muscles were immunostained with anti-α-bungarotoxin tetramethylrhodamine conjugate. The entire length of the muscle in the same level of cross-section was evaluated under confocal microscope and acquired in a mosaic pattern. The neuromuscular junctions were counted in the ipsilateral and contralateral sides 5 weeks after peripheral nerve injury. Such numbers were obtained by using Image Tool software (University of Texas, USA).

### Transmission electron microscopy

After fixation, the contralateral and crushed sciatic nerves from each animal (*n* = 5 for each group) were osmicated, dehydrated and embedded in Durcupan ACS (Fluka, Steinheim, Switzerland). Ultrathin cross sections obtained from the distal stump (2.0 mm distally to the lesion site) and from the collateral side. They were collected on formvar-coated copper grids, contrasted with uranyl acetate and lead citrate, and examined under a Spirit BioTwin G2 (FEI Company, The Netherlands) transmission electron microscope (TEM) operating at 120 KV. All measurements were performed in a set of images corresponding to 50 % of the total nerve area, obtained automatically with a photomontage software (FEI Company, The Netherlands). The number of myelinated and degenerating fibers and non-myelinated axons were acquired manually using the Image Tool software (University of Texas, USA).

Morphometric analysis of axons in the ipsi and contralateral sides was performed to evaluate the quality of nerve regeneration. Therefore, diameter of myelinated fibers (D) were obtained from the values of their perimeters (P) applying the formula *D* = *P*/*π*. Also, the thickness of the myelin sheath [TMS] was calculated by the difference between the diameter of myelinated fibers [DMF] and diameter of myelinated axons [DMA] divided by 2, according to [15].

### Motor function recovery

For the gait recovery analysis, the CatWalk system (Noldus Inc, The Netherlands) was used. In this method, the animal crosses a walkway with a glass floor illuminated from the long edge. Data acquisition was carried out by a high-speed camera and the paw prints were automatically classified by the software. The paw prints from each animal (minimum of *n* = 8 per group) were obtained before and after the sciatic crushing. Post-operative data were assessed on the third, fourth and fifth days following surgical intervention, and then twice a week until 8 weeks post-lesion.

The parameters used herein to calculate the sciatic functional index were the distance between the third toe and hind limb pads (print length, PL), and the distance between the first and fifth toes (toe spread, TS). Measurements of the parameters were obtained from the right (normal) and left (experimental) paw prints and the values were used to calculate the sciatic functional index (SFI), according to the following formula [16]: SFI = 118.9 (ETS - NTS/NTS) - 51.2 (EPL - NPL/NPL) - 7.5 (E = experimental side, N = normal side). In addition, the pressure exerted by the individual paws during contact with the platform was evaluated. The data were expressed as the lesioned/unlesioned ratio for each day of training.

### Statistical analysis

The data are presented as mean ± SEM, and the differences between groups were considered significant when the *P* value was <0.05 (*). Statistical analysis was performed with GraphPad Prisma 4.0 software. In this sense, data were subjected to ANOVA followed by Bonferroni post hoc test for parametric data or Mann-Whitney *U* test for non-parametric data.

## Results

### TLR4 mutant mice presented earlier motor recovery with correlative increase of p75NTR in the distal nerve stump without changes in macrophage recruitment/activation

**Fig. 1:**
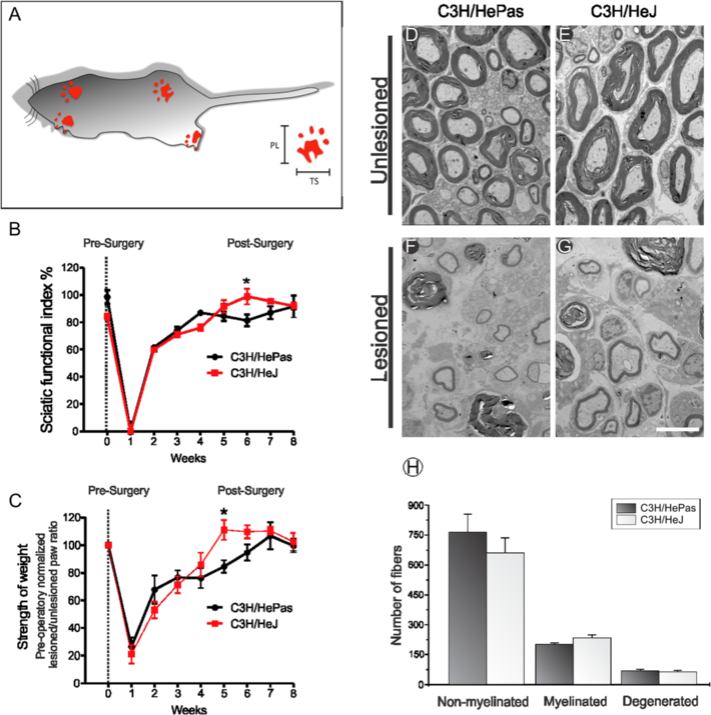
**a** Schematic drawing representing the mice paw prints under CatWalk system. Measurements used to calculate the sciatic functional index: distance between the first and fifth toes (TS- Toe spread) and third toe and hind limb pads (PL print length). **b, c** Cat walk analysis in crush-lesioned C3H/HePas and C3H/HeJ mice. The paw prints from each animal were obtained before and after the sciatic crush. **b** Graph of the sciatic functional index (SFI) values according to the group and time of evaluation. **c** The intensity of the paw print is shown as a percentage of the contralateral paw during 8 weeks post-surgery. The data are presented as the percentage of the pre-surgery values. **d**–**g** Ultrastructure images of the sciatic nerve from C3H/HePas and C3H/HeJ groups. **d**, **e** Contralateral and (**f** and **g**) ipsilateral stumps of sciatic nerves in wild-type and mutant mice. **h** Graph of the number of myelinated, unmyelinated, and degenerated fibers. *Scale bar*: 5 μm

Based on our previous published data that shown TLR4 contributes to synaptic stabilization on motoneurons after the peripheral nerve lesion [12], we expected finding a less successful functional recovery in the TLR4 mutant mice. Contrarily, the present results revealed that mutant group supported more weight on ipsilateral paw compared to the wild type. Five weeks after surgery, the mutant mice presented an earlier functional recovery evidenced by strength of paw weight (C3H/HePas: fifth week 84.58 % ± 4.16 %; C3H/HeJ: fifth week 110.97 % ± 6.46 %; Fig. 1c). Following 6 to 8 weeks the sciatic functional index shows significant difference between groups (C3H/HePas: sixth week 81.23 % ± 4.29 %; seventh week 87.03 % ± 4.73 %; eighth week 91.53 % ± 7.89 %; C3H/HeJ sixth week 98.73 % ± 5.50 %; seventh week 95.39 % ± 1.44 %; eighth week 91.75 % ± 2.75 %, Fig. 1b).

**Fig. 2:**
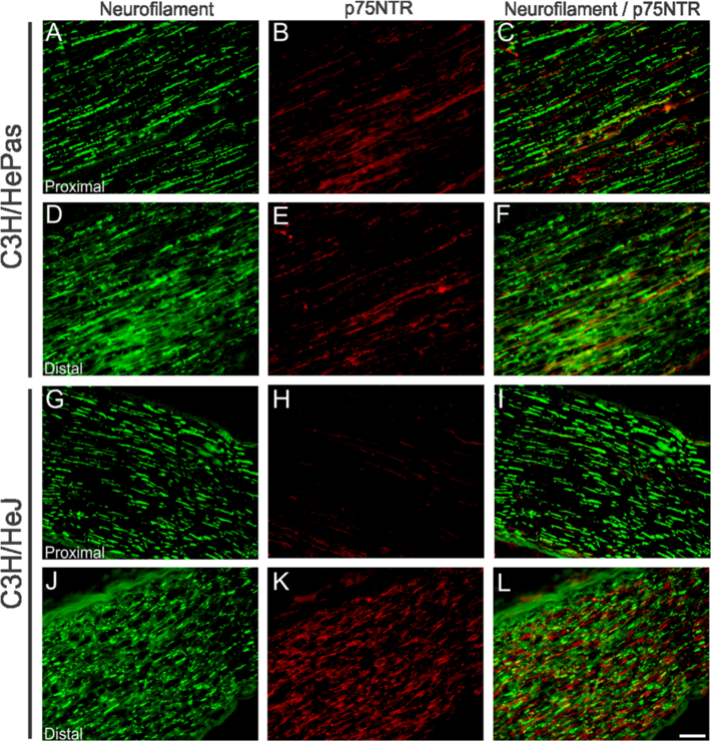
Representative images of neurofilament and p75NTR immunostaining in C3H/HePas and C3H/HeJ mice 2 weeks after unilateral crush. C3H/HePas proximal stump (**a**–**c**) and C3H/HeJ (**g**–**i**). C3H/HePas distal stump (**d**–**f**) and C3H/HeJ (**j**–**l**). Note that the neurofilament and p75NTR immunolabeling is more intense in the distal stump of mutant mice compared to the wild type. *Scale bar* = 50 μm

Immunolabeling of neurofilament and p75NTR protein showed that the greater functional recovery in mutante mice TLR4 was correlated with a higher expression of p75NTR and neurofilament protein expression in the distal stump that indicate an augmented ability to axonal regrowth or regeneration (Fig. 2).

To further confirm whether mutant mice TLR 4 present an augmented number of regenerated fibers, ultrastructural analysis were performed in non-myelinated axons, myelinated fibers, and those undergoing degenerative processes 2 weeks after crushing. Morphometric analysis of myelinated fibers, axons, and thickness of the myelin sheath also was evaluated in the ipsilateral and contralateral sides to the lesion in C3H/HePas and C3H/HeJ mice (Additional file 1: Figure S1). We found that the better functional recovery in mutant mice was not a result of changes in morphometric parameters of myelinated axons (C3H/HePas 765.00 ± 88.73; 215.80 ± 6.81; 70.00 ± 7.23, C3H/HeJ 660.00 ± 73.94; 233.00 ± 16.26; 63.60 ± 5.24 number of unmyelinated, myelinated, and degenerated fibers, respectively, ±standard error; Fig. 1h).

**Fig. 3:**
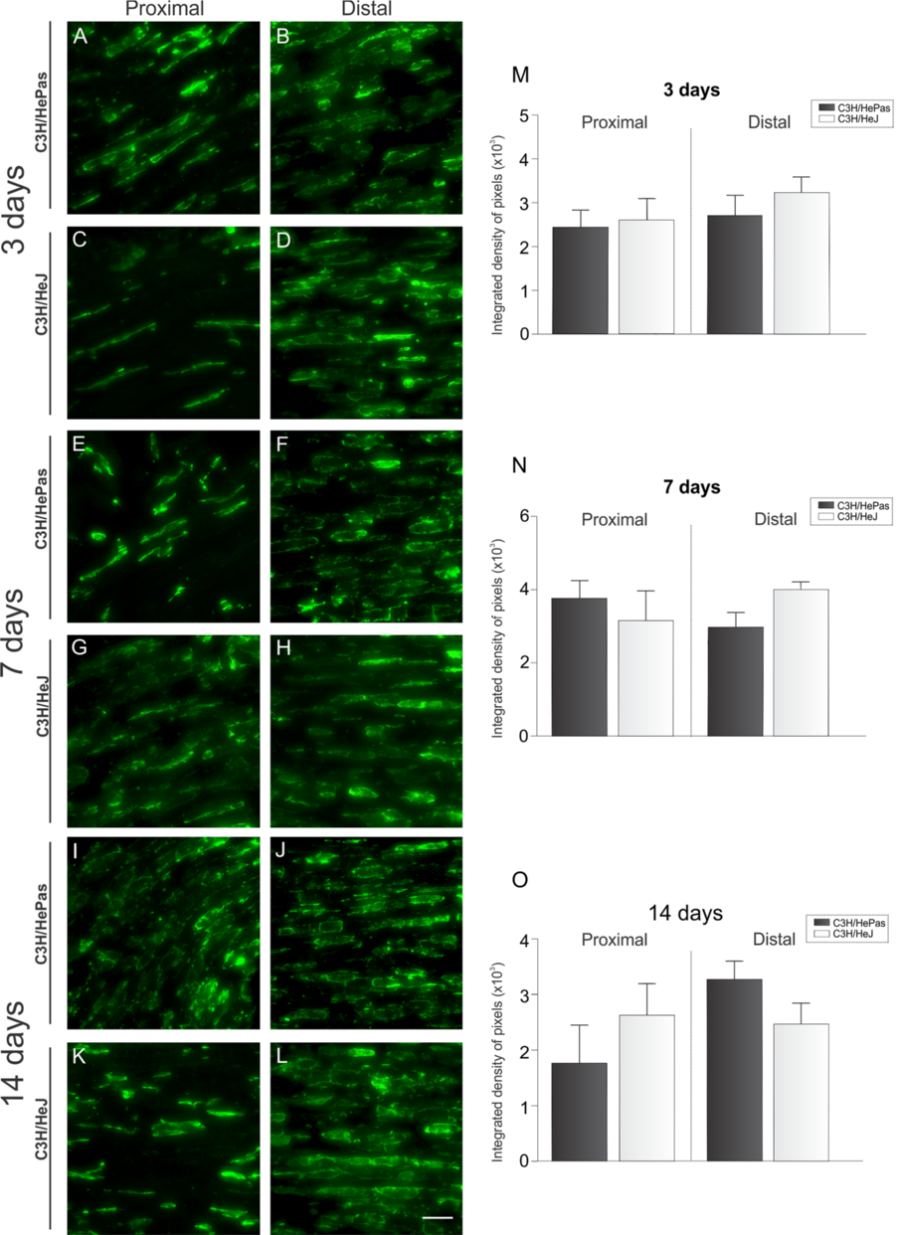
Representative images of macrophages activation in longitudinal sections of the proximal and distal stumps of sciatic nerve. Immunolabeling was performed in C3H/HePas and C3H/HeJ mice at 3 (**a**–**d**), 7 (**e**–**h**) and 14 (**i**–**l**) days after nerve crush, times that correspond to key steps in the remyelination process. (**m**–**o**) Graphs of the integrated density of pixels of activated macrophages under an equivalent area for all groups. Note that the absence of TLR4 did not influence the recruitment of macrophages to the lesion site at any time analyzed. *Scale bar*: 30 μm

In addition, the better functional recovery in mutant mice was not a result of changes in macrophage recruitment/activation as shown in Fig. 3. Iba-1 immunolabeling shows no difference between both strains at 3, 7, and 14 days after nerve crushing at either proximal or distal stump. The integrated density of pixels (×10^3^) for proximal stump were at 3 days: C3H/HePas - 2.42 ± 0.41; C3H/HeJ - 2.59 ± 0.50; 7 days: C3H/HePas - 3.77 ± 0.49; C3H/HeJ - 3.16 ± 0.82 and; 14 days: C3H/HePas - 1.76 ± 0.69; C3H/HeJ - 2.63 ± 0.59 (Fig. 3). In addition, the values for the distal stump were at 3 days: C3H/HePas - 2.71 ± 0.47; C3H/HeJ - 3.25 ± 0.37; 7 days: C3H/HePas - 2.92 ± 0.42; C3H/HeJ - 3.96 ± 0.21; 14 days: C3H/HePas - 3.25 ± 0.33; C3H/HeJ - 2.44 ± 0.38 (Fig. 3).

### Deficiency of TLR4 leads to an increased number of neuromuscular junctions

**Fig. 4:**
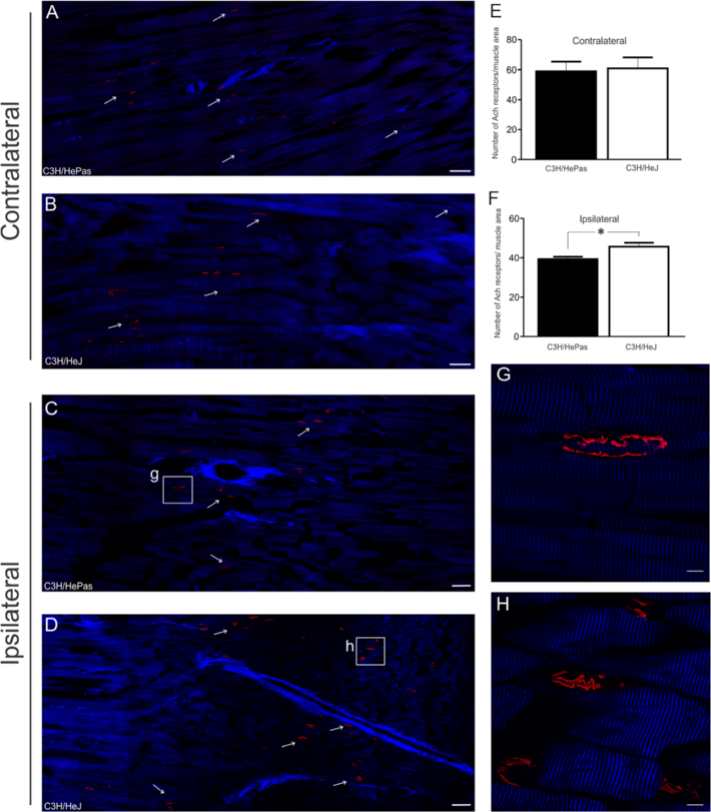
Representative images of tibialis cranialis muscle in the horizontal plane showing a greater number of neuromuscular junctions in mutant mice. Neuromuscular junctions immunostained with anti-α-bungarotoxin tetramethylrhodamine conjugate (*red*). Muscle myofibrils (*blue*) by using optical system second harmonic generation (SHC). (**a**–**b**) Contralateral and (**c**–**d**) ipsilateral sides of tibialis cranialis muscle, respectively, in C3H/HePas and C3H/HeJ mice. (**e**–**f**) Graphs of the number of neuromuscular junctions per muscle area. (**g**–**h**) Neuromuscular junctions immunostained in details. Note that the functional recovery observed in mutant mice might be a result of an increase of nerve terminal sprouts. *Scale bar*: 100 μm

Although the morphometrical results did not show increased number or improved thickness of axons, a greater number of neuromuscular junctions have been detected in the tibialis cranialis muscle, by immunolabeling with anti-α-bungarotoxin. Such analysis was performed in both strains (TLR 4 mutant and wild type) at 5 weeks post-injury. This particular time point was chosen based on the observation of improvement of functional recovery. Accordingly, our data show that TLR4 mutant faster improvement was due to a greater number of neuromuscular sprouts, which led to a 20 % increase in the number of neuromuscular junctions at the tibialis cranialis muscle (C3H/HePas - 38.17 ± 1.42; C3H/HeJ - 45.67 ± 1.96; Fig. 4). No difference was observed in the contralateral side, confirming that the difference between groups occurs during recovery after peripheral nerve injury (C3H/HePas 59.03 ± 6.30; C3H/HeJ 60.83 ± 7.31; Fig. 4).

### Absence of TLR2 does not influence gait recovery and macrophage recruitment, but leads to greater degeneration in the distal stump combined with decreased p75NTR expression

**Fig. 5:**
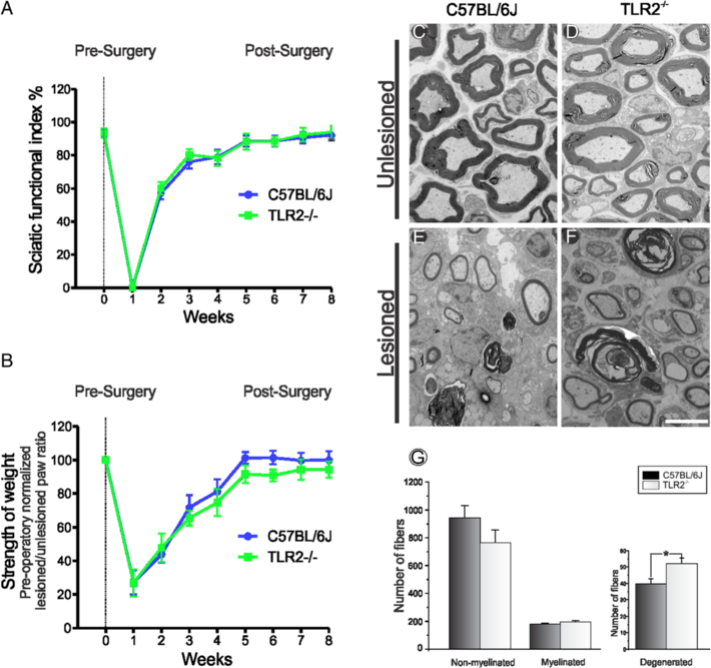
**a**, **b** Gait analysis after sciatic nerve crush in C57BL/6 J and TLR2^−/−^ by using the CatWalk. The paw prints from each animal were obtained before and after the sciatic crush. **a** Graph of the sciatic functional index (SFI) shows the evaluation of motor recovery up to 8 weeks post-lesion in the experimental groups C57BL/6 J and TLR2^−/−^. **b** The intensity of the paw print shows the hind limb paw pressure in both experimental groups. **c**–**f** Ultrastructure images of the sciatic nerve from C57BL/6 J and TLR2^−/−^ groups. **g** Graph of the number of myelinated, unmyelinated and degenerated fibers. Note a greater number of degenerated fibers in knockout mice. *Scale bar*: 5 μm

Evaluation of motor function in TLR2^−/−^ mice after sciatic nerve crush showed that absence of TLR 2 does not influence the overall functional recovery (Fig. 5a, b). Further, we performed morphometric analysis of myelinated fibers and thickness of myelin sheath in the ipsilateral and contralateral sides to the lesion in TLR2^−/−^ and wild-type mice (Additional file 2: Figure S2).

Ultrastructural analysis of number of unmyelinated, myelinated, and degenerated axons was performed at the sciatic nerve mid-thigh in the ipsi- and contralateral sides in all groups. Knockout mice for TLR2 showed 30 % greater number of degenerated axons in the distal nerve stump when compared to the wild type (C57BL/6 J - 198.86 ± 88.93; 177.8 ± 5.84; 39.40 ± 3.28; TLR2^-/-^ - 207.73 ± 92,89; 193.20 ± 7.97; 52.00 ± 3.38; number of unmyelinated, myelinated, and degenerated fibers, respectively, ± standard error; Fig. 5c–g).

**Fig. 6:**
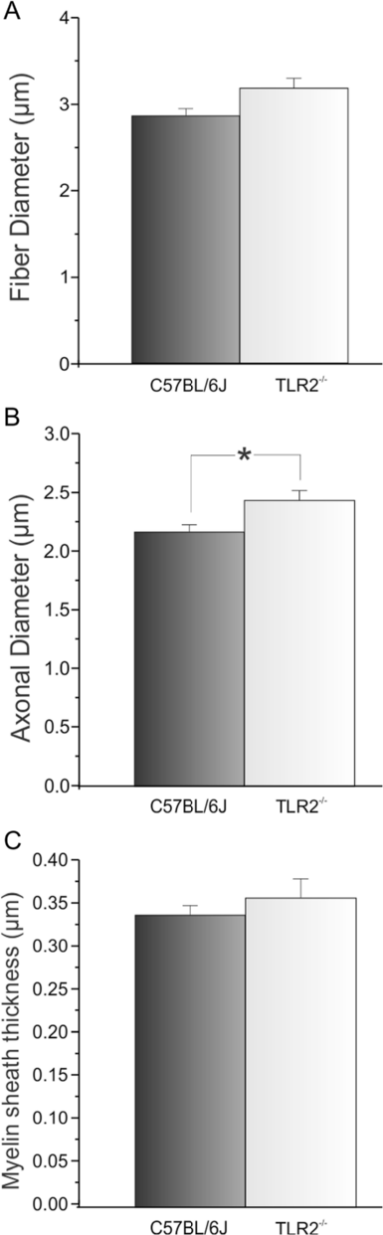
Morphometric analysis of fiber diameter (**a**), axonal diameter (**b**) and myelin sheath thickness (**c**) in C57BL/6 J and TLR2^−/−^ groups after nerve crush

**Fig. 7:**
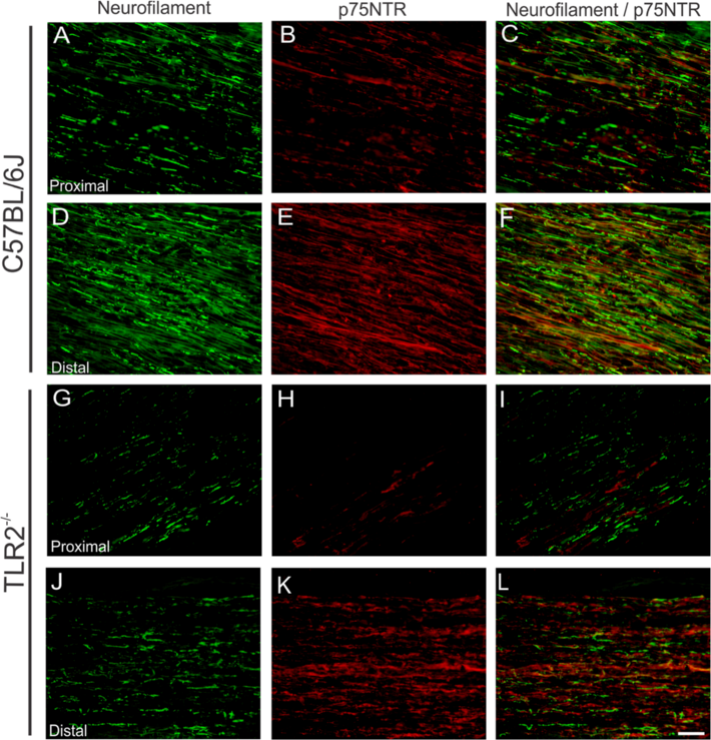
Representative images of neurofilament and p75NTR immunostaining in C57BL/6 J and TLR2^−/−^ mice 2 weeks after unilateral sciatic nerve crush. C57BL/6 J proximal stump (**a**–**c**) and TLR2^−/−^ (**g**–**i**). C57BL/6 J distal stump (**d**–**f**) and TLR2^−/−^ (**j**–**l**). Note that p75NTR immunolabeling is less intense in the proximal stump of mutant mice compared to the wild type. *Scale bar*: 50 μm

Curiously, TLR2^-/-^ showed a significant increase in the mean diameter of myelinated axons, two weeks after injury as compared to the wild-type mice (fiber diameter - μm: C57BL/6 J - 2.86 ± 0.08; TLR2^−/−^ - 3.18 ± 0.11; axon diameter: C57BL/6 J - 2.17 ± 0.06; TLR2^−/−^ - 2.44 ± 0.08; thickness of myelin sheath: C57BL/6 J - 0.34 ± 0.01; TLR2^−/−^ - 0.37 ± 0.02 Fig. 6). Moreover, TLR2^−/−^ exhibited decreased p75NTR and neurofilament protein expression (Fig. 7).

**Fig. 8:**
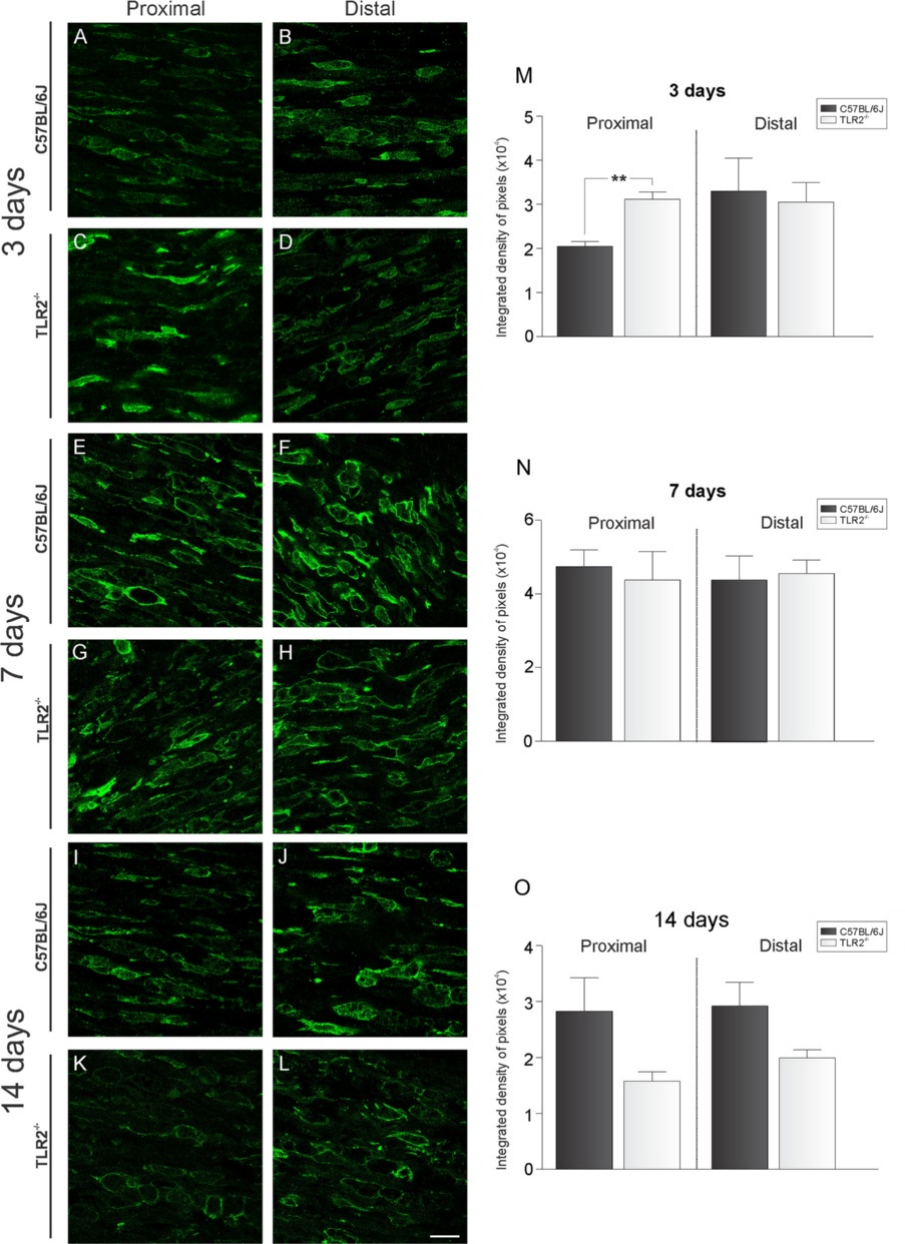
Representative images of macrophages activation in longitudinal sections of the proximal and distal stumps of sciatic nerve. Immunolabeling was performed in C57BL/6 J and TLR2^−/−^ mice at 3 (**a**–**d**), 7 (**e**–**h**) and 14 (**i**–**l**) days after nerve crush. (**m**–**o**) Graphs of the integrated density of pixels of activated macrophages under an equivalent area for all groups. Note that de absence of TLR2 does not influence the recruitment of macrophages to the lesion site. *Scale bar*: 50 μm

Additionally, we further determined whether the occurrence of greater number of degenerated fibers in the distal stump was due to a delay in the macrophage recruitment/activation. For that purpose, immunolabeling of Iba-1 was performed in both strains at 3, 7, and 14 days after nerve crushing. The results indicate that TLR2^−/−^ does not present a delay or deficiency on macrophage recruitment/activation compared to wild-type mice. Contrarily, there was a significantly increase of macrophages at proximal stump 3 days after peripheral nerve injury (proximal stump – 3 days: C57BL/6 J – 2.05 ± 0.18; TLR2^-/-^ - 3.12 ± 0.16; 7 days: C57BL/6 J – 4.75 ± 0.42; TLR2^−/-^ - 4.38 ± 0.78; 14 days: C57BL/6 J – 2.82 ± 0.59; TLR2^−/−^ - 1.57 ± 0.17; distal stump- 3 days: C57BL/6 J – 3.31 ± 0.74; TLR2^−/−^ - 3.05 ± 0.43 7 days: C57BL/6 J – 4.39 ± 0.66; TLR2^−/−^ - 4.55 ± 0.36; 14 days: C57BL/6 J – 2.92 ± 0.39; TLR2^−/−^ - 1.93 ± 0.21; Integrated density of pixels (×10^4^) ± SE; Fig. 8).

### Absence of TLR2 does not influence the number of neuromuscular junctions following sciatic nerve regeneration

**Fig. 9:**
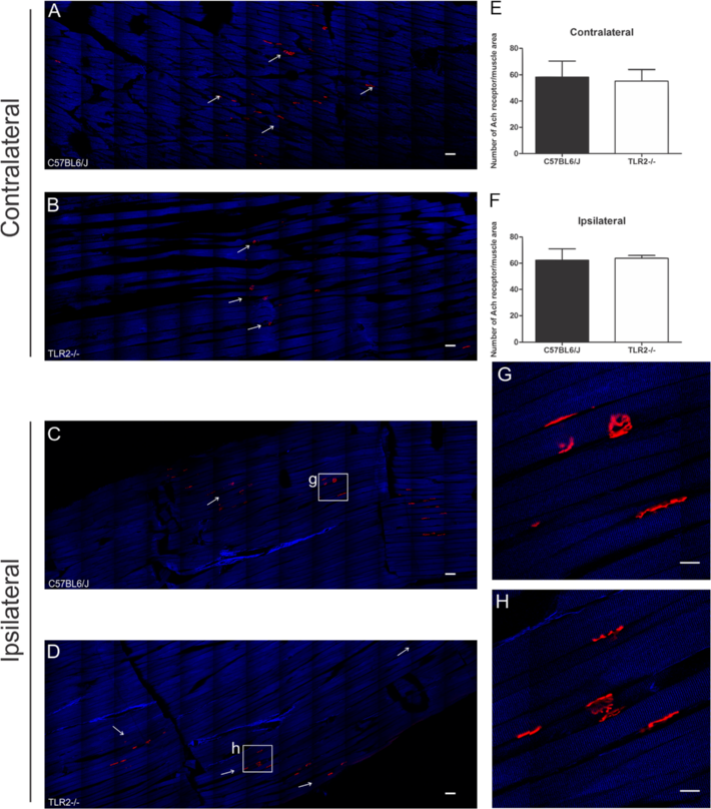
Representative images of tibialis cranialis muscle in the horizontal plane showing the neuromuscular junctions in C57BL/6 J and TLR2^−/−^ mice. Neuromuscular junctions immunostained with anti-α-bungarotoxin tetramethylrhodamine conjugate (*red*). Muscle myofibrils (*blue*) by using optical system second harmonic generation (SHC). (**a**–**b**) Contralateral and (**c**–**d**) Ipsilateral sides of tibialis cranialis muscle, respectively, in C57BL/6 J and TLR2^−/−^ mice. *Scale bar*: 300 μm. (**e**–**f**) Graphs of the number of neuromuscular junctions per muscle area. (**g**–**h**) Neuromuscular junctions immunostained in details. *Scale bar*: 100 μm

Given that both strains presented equal gait recovery, we evaluated the number of neuromuscular junctions present in the tibialis cranialis muscle. Likewise, in TLR4 groups, alpha-bungarotoxin labeling was performed in TLR2-/- and C57BL/6 J mice. The survival time of 5 weeks post sciatic nerve crushing was chosen based on the fact that both groups achieved more than 80 % motor recovery at that point. No differences were found regarding the number of neuromuscular endplates (contralateral: C57BL/6 J 58.22 ± 12.07; TLR2^−/−^ 55.25 ± 8.74; ipsilateral: C57BL/6 J 62.28 ± 8.66; TLR2^−/−^ 63.78 ± 2.09 Fig. 9).

## Discussion

Early response to peripheral nerve injury involves degeneration of axons and myelin sheath in the distal stump, as well as retrograde changes at neuronal cell body in the spinal cord and dorsal root ganglia. Degenerated myelin and nerve debris are both major inhibitors to regeneration so that tissue clearance is of substantial importance to allow timely and precise axonal regeneration distally to injury. Phagocytosis is carried out by Schwann cells and monocyte-derived macrophages, so that prompt activation is necessary [2]. Nevertheless, the precise mechanisms for such recruitment ought to be clearly understood.

The interaction between nervous and immune system has become a strategic field of research, since there is a clear overlap of molecules and cellular crosstalk that may become new therapeutic targets. In this regard, toll-like receptors (TLR) are strongly activated in the traumatized peripheral nerve microenvironment, which might be related to the response to injury, including monocyte diapedesis, Schwann cell proliferation and secretion of neurotrophic factors, as well as phagocytic activity [17]. Boivin et al. [11] showed that significantly fewer macrophages were recruited and/or activated in the distal stump of sciatic nerve upon crush injury in TLR2^−/−^, TLR4^−/−^ and MyD88 deficient mice. In addition, they showed that a microinjections of TLR2 and TLR4 ligands at the site of sciatic nerve lesion result in faster clearance of the degenerating myelin and earlier recovery.

The results herein, however, differ from the reported by Boivin et al. [11]. In this sense, the absence of TLR2 resulted in macrophage labeling increase in the proximal stump of the crushed nerve at 3 days after injury. However, no difference between knockout (TLR2-KO and TLR4-mutant) and wild-type macrophages recruitment could be seen at 7 and 14 days after injury. Such result indicates that greater number of degenerated fibers in the distal stump is not due to decreased macrophage recruitment/activation.

One important factor to be emphasized, regarding PNS response to injury, that may in turn result in conflicting results is the extension of the lesion itself. Experimentally, different approaches have been used, including neurotmesis (complete nerve transection) followed by neurorrhaphy (nerve stumps coaptation), crush injury, as well as the microcrush lesion. The microcrush injury, that is carried out by tying a knot (that is released after 60 s) to constrict the nerve and thus to generate axotomy, may generate divergent data when compared to a broader crushing injury. In this scenario, the blood nerve barrier is minimally affected, contrarily to the present work, which employed a larger crush injury, facilitating migration of blood-born cells to the territory of the lesion. In this sense, we observed similar blood-born macrophage influx to the lesioned nerve area in mutant and wild-type mice. Contrarily, by microcrushing the nerve, Boivin et al. [11], reported decreased macrophage migration in Myd88, TLR2-KO, and TLR4-mutant.

In our study, mice with absence of functional TLR4 showed better function recovery correlated with increased number of neuromuscular junctions at the tibialis cranialis muscle. Such greater number of neuromuscular junctions matches with the earlier increase of neurotrophin receptor p75NTR in the distal nerve stump, suggesting greater capability to terminal axon sprouting. Recent study showed that blocking the receptor of p75NTR which is present in the nerve terminal, muscle cell and glial Schwann cell at the neuromuscular junction, result in reduced synapse function at neuromuscular junction [18]. Moreover, it is well-known that p75NTR expression in the Schwann cells stimulate axonal growth and remyelination with consequent improvement of locomotor recovery [19].

Recent data on CNS/PNS response to injury and inflammatory mechanisms firmly indicates that molecules from the immune system may develop particular roles in the nervous system microenvironment. MHC of class I has been shown during visual system development [20] and after adult peripheral nerve lesion [21–23]. Additionally, other immune-related molecules from the complement system have also been implicated in synaptic plasticity events. Due to the inflammatory events that take place in the peripheral nerve following injury, toll-like receptors are also candidate molecules that may develop additional roles apart from the already known mechanisms of pathogen recognition. In this way, we have recently shown that absence of TLR2 and of functional TLR4 results in spinal cord changes following peripheral nerve lesion, affecting dynamics of synapses related to spinal motoneurons [12]. Taking into account that TLRs are directly involved in myelin clearance during WD, it is plausible to assume possible roles in the regeneration process.

In the present study, motor recovery was evaluated with an automated walking track system, based on the capture of illuminated paw prints (Catwalk system, Noldus Inc., The Netherlands). Such technology allows calculation of integrated density of pixels and, in turn, to obtain pressure values. This is particularly interesting since it makes possible to evaluate strength as well as gait recovery parameters. Based on such technology, it was possible to demonstrate that the absence of functional TLR4 increases the strength recovery, from the fifth-week post-lesion, as compared to wild-type animals. Nevertheless, the sciatic functional index remains similar between strains. Such data is in line with the greater amount of neuromuscular junctions seen in TLR4 mutant mice and suggest the existence of compensatory mechanisms that counteract the absence of poor inflammatory signaling during WD [7, 24]. The use of a more sensitive device to record gait recovery may have also contributed to the differences in comparison to the one reported by [11].

We have recently demonstrated that toll-like receptors 2 and 4 have opposite roles on synaptic stability in the spinal cord after peripheral nerve injury [12]. It is well-established that neurons suffer retrograde metabolic and morphological changes in response to axotomy, which may influence the subsequent regeneration process [25, 26]. The lesion itself also stimulates the proliferation of Schwann cells, influencing the interaction between regenerating axons and glial cells [27]. Accordingly, better preservation of spinal cord microenvironment would positively influence distal axonal regrowth, as suggested in TLR2^−/−^ [12]. However, evaluation of motor function in TLR2^−/−^ mice after crushing showed absence of overall functional recovery difference in comparison to the wild-type counterpart. In addition, TLR2^−/−^ mice presented more degenerating axons and myelin debris, suggesting a faster phagocytosis in such transgenic strain, since we showed augmented recruitment of macrophages at distal stump 3 days after the injury. However, no macrophage changes could be depicted in mutant mice 7 and 14 days post-lesion, opposing to [11]. As already discussed, the evidence of strikingly similar motor recovery between TLR2^−/−^ and wild-type animals suggest that compensatory mechanisms in these mice may occur such as reported thicker axonal diameter. Studies showed that increase in diameter of axons are correlated to better functional recovery [28, 29].

## Conclusions

Taken together, the results of the present work indicate, for the first time, that deficiency or absence of TLR2 and of functional TLR4 may be counteracted by compensatory mechanisms that result in normal axonal regeneration and functional recovery. This may in turn contribute to the development of new immunomodulatory treatment approaches resulting in improved regeneration and functional recovery.

## Ethics approval

The Institutional Committee for Ethics in Animal Use approved the study (CEUA/IB/Unicamp, no. 1656-1 and 2104-1), and the experiments were carried out in accordance with the guidelines of the National Council for the Control of Animal Experimentation (CONCEA).

## Consent for publication

Not applicable.

## Availability of data and materials

The dataset supporting the conclusions of this article is available in the following link: https://www.dropbox.com/sh/v1qa1aybm6nesj9/AAC7Q1mmPaHSFZXRPeVPjn8ya?dl=0

## Additional files

Additional file 1: Figure S1. Morphometric analysis of myelinated axons (A-L). Graphs of the frequency distribution of myelinated axons in C3H/HePas (A-C before injury) and (D-F after the injury); C3H/HeJ (G-I before injury) and (J-L after the injury). Note that functional recovery was not the result of an increase in number or thickness of myelinated axons. (TIF 435 kb) Additional file 2: Figure S2. Morphometric analysis of myelinated axons (A-L). Graphs of the frequency distribution of myelinated axons in C57BL/6 J (A-C before injury) and (D-F after the injury); Knockout (TLR2-/-) (G-I before injury) and (J-L after injury). Note an increased in the diameter of myelinated axons after injury, which may have contributed to a similar locomotor performance in both groups. (TIF 424 kb)

## Abbreviations

DMA: diameter of myelinated axons
iNOS: inducible nitric oxide synthase
PB: phosphate buffer
PL: print length
PNI: peripheral nerve injury
SFI: sciatic functional index
SHC: second harmonic generation
TEM: transmission electron microscope
TLRs: toll-like receptors
TMS: thickness of the myelin sheath
TNF-α: tumor necrosis factor alpha
TS: toe spread
WD: Wallerian degeneration
