# Supplementary figures and images for: Impairment of toll-like receptors 2 and 4 leads to compensatory mechanisms after sciatic nerve axotomy
Source: J Neuroinflammation. 2016 May 24;13:118. doi: 10.1186/s12974-016-0579-6 (PMC4879730; doi:10.1186/s12974-016-0579-6)

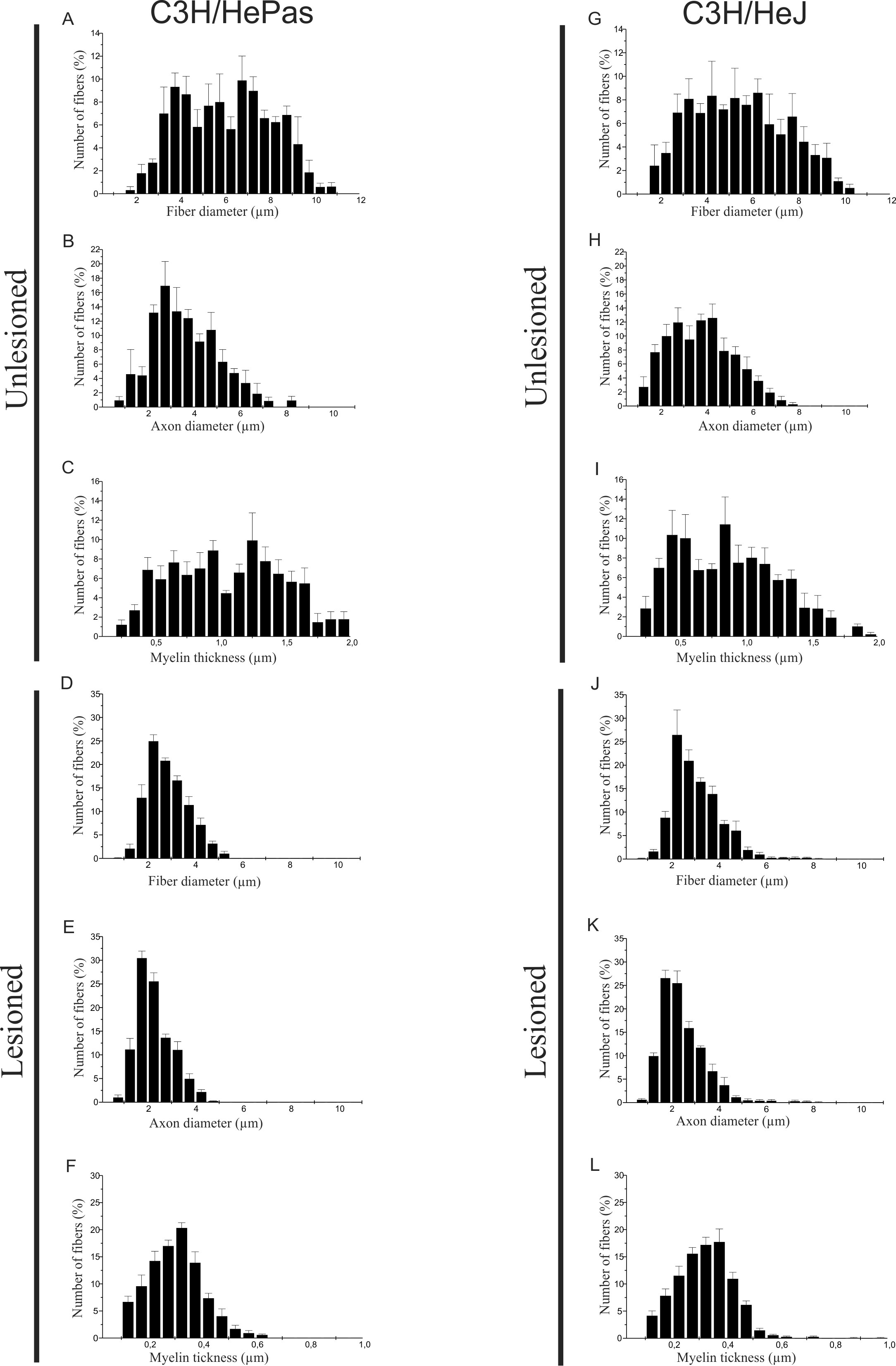

Supplement: Additional file 1: Figure S1. — Morphometric analysis of myelinated axons (A-L). Graphs of the frequency distribution of myelinated axons in C3H/HePas (A-C before injury) and (D-F after the injury); C3H/HeJ (G-I before injury) and (J-L after the injury). Note that functional recovery was not the result of an increase in number or thickness of myelinated axons. (TIF 435 kb) [file 12974_2016_579_MOESM1_ESM.tif]

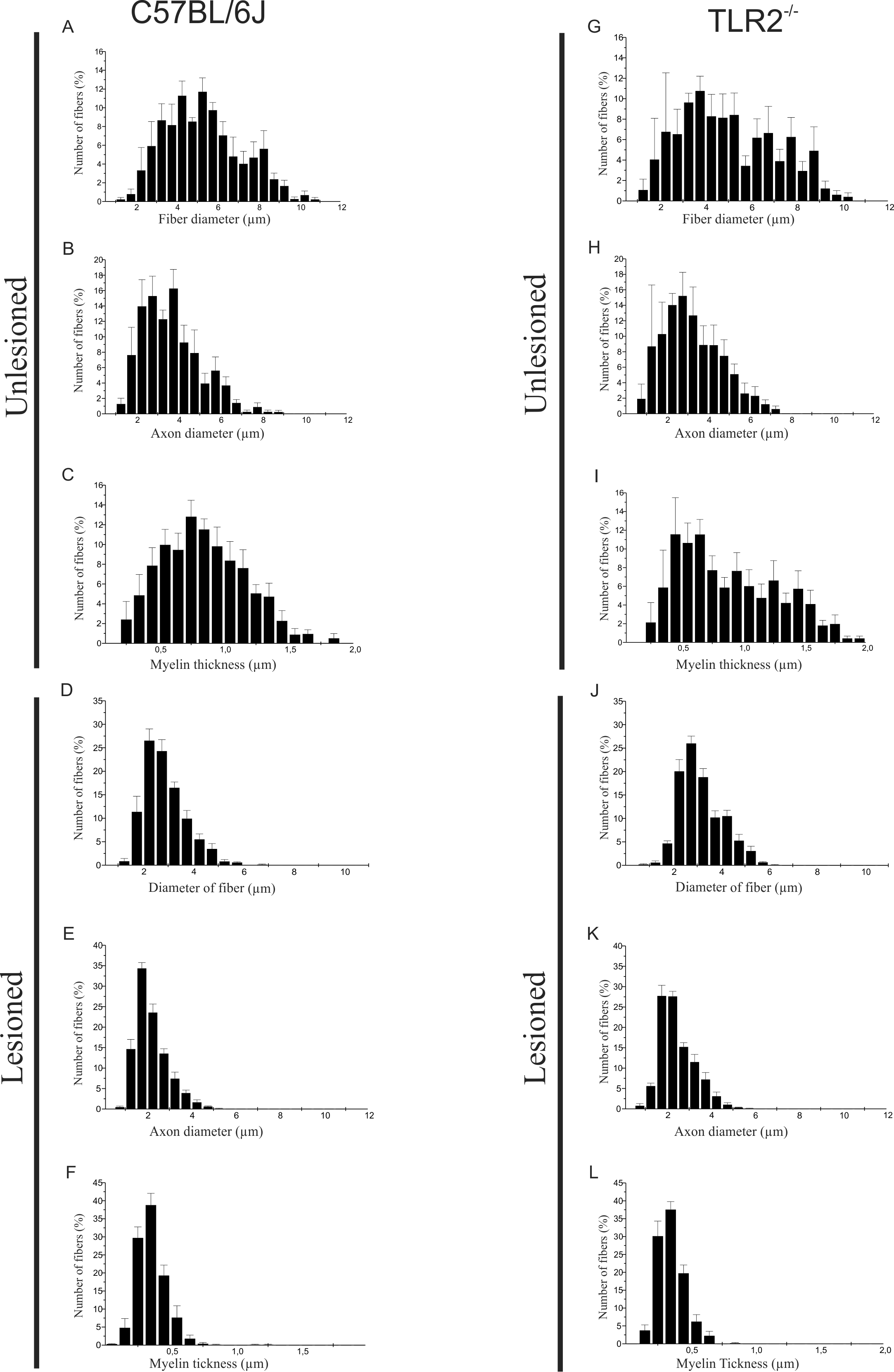

Supplement: Additional file 2: Figure S2. — Morphometric analysis of myelinated axons (A-L). Graphs of the frequency distribution of myelinated axons in C57BL/6 J (A-C before injury) and (D-F after the injury); Knockout (TLR2-/-) (G-I before injury) and (J-L after injury). Note an increased in the diameter of myelinated axons after injury, which may have contributed to a similar locomotor performance in both groups. (TIF 424 kb) [file 12974_2016_579_MOESM2_ESM.tif]
